# Comparing the HER2 Status of the Primary Tumor to That of Disseminated Tumor Cells in Early Breast Cancer

**DOI:** 10.3390/ijms25115910

**Published:** 2024-05-29

**Authors:** Léa Louise Volmer, Dominik Dannehl, Sabine Matovina, Florin-Andrei Taran, Christina Barbara Walter, Markus Wallwiener, Sara Yvonne Brucker, Andreas Daniel Hartkopf, Tobias Engler

**Affiliations:** 1 Department of Women’s Health, Tübingen University, 72076 Tübingen, Germany; dominik.dannehl@med.uni-tuebingen.de (D.D.); sabine.matovina@med.uni-tuebingen.de (S.M.); christina-barbara.walter@med.uni-tuebingen.de (C.B.W.); sara.brucker@med.uni-tuebingen.de (S.Y.B.); andreas.hartkopf@med.uni-tuebingen.de (A.D.H.); tobias.engler@med.uni-tuebingen.de (T.E.); 2 Department for Gynecology and Obstetrics, Freiburg University, 79085 Freiburg im Breisgau, Germany; florin-andrei.taran@uniklinik-freiburg.de; 3 Department for Gynecology and Obstetrics, University Medical Center Halle, 06120 Halle (Saale), Germany; markus.wallwiener@uk-halle.de

**Keywords:** disseminated tumor cells, minimal residual disease, breast cancer, targeted therapy

## Abstract

Breast cancer remains a leading cause of cancer mortality in women globally. Despite advancements in systemic therapy, the risk of distant recurrence persists even after such treatment and may be linked to disseminated tumor cells (DTCs). Variability in molecular characteristics between primary tumors (PTs) and distant metastases underscores the need to comprehensively understand metastatic pathways. This retrospective study investigated discrepancies between HER2 expression in PTs and DTCs and their implications for survival outcomes in 201 early breast cancer (EBC) patients. We found a significant association between HER2 expression in PTs and DTCs when classifying tumors as HER2-high/low/negative. Patients whose HER2 status was discordant between PTs and DTCs exhibited worse distant disease-free survival than those with concordant status. Multivariate analysis confirmed the HER2 status of DTCs as an independent prognostic factor for distant DFS. These findings emphasize the importance of assessing HER2 expression in DTCs and its potential implications for tailored therapy strategies in EBC. Furthermore, prospective trials are needed to validate these findings and explore targeted therapies based on the molecular characteristics of DTCs.

## 1. Introduction

Breast cancer is the most frequently diagnosed cancer and leading cause of cancer death among women worldwide [1,2], with increasing incidences in a large number of populations [3]. Throughout the last few decades, clinical outcome and prognosis of breast cancer patients could be improved thanks to the emergence of new options for systemic therapy [4,5]. However, the disease may still recur at distant sites even years after primary therapy, despite optimal surgical and systemic treatment [6]. A possible explanation for distant breast cancer recurrence is minimal residual disease (MRD), where single tumor cells spread even in the earliest stages of breast cancer [7]. Disseminated tumor cells (DTCs) represent one manifestation of MRD: these cells can be detected in the bone marrow (BM) of 20–30% of patients with early breast cancer (EBC) [8]. Indeed, the detection of DTCs is associated both with a worse outcome and with locoregional and distant recurrence alike [9,10,11].

Previous studies showed that molecular characteristics can vary between the primary tumor (PT) and distant metastases [12,13,14]. More specifically, discrepancies in HER2 expression between the PT and metastases could be shown in almost 50% of cases [15,16]. In addition to tumor plasticity [17], another explanation for these differences may be that early spreading cells differ from the PT or that specific subclones from the PT have a higher tendency to metastasize [18,19]. For example, differences between the PT and DTCs could be shown in regard to HER2 status [20]. Therefore, DTCs expressing different molecular features may escape therapy for EBC, which is based solely on PT characteristics.

With the emergence of new targeted therapies against breast cancer, new approaches to interpreting molecular characteristics have been developed, too [21]. Not only have new targets such as TROP2 been discovered but classical molecular parameters have also been revisited [22]. Since the new substance trastuzumab deruxtecan was shown to be effective in breast cancer tumors with “low” HER2 expression [23], more attention has been paid to breast cancer cases with a 1+ or 2+ immunohistochemical (IHC)-HER2 expression [24].

An association between the HER2 status of DTCs and survival in EBC was shown in a previous study, where the presence of HER2-positive DTCs was associated with a poorer disease-free survival (DFS) [20]. The aim of the present study was to evaluate a possible association between HER2 expression in the PT and DTCs under the actualized context in interpreting HER2 expression. Furthermore, it was the goal of this work to analyze possible implications of the HER2 status of DTCs for survival and follow-up under a changed therapy landscape.

## 2. Results

### 2.1. Patient Characteristics

A total of 201 EBC patients were eligible for this retrospective analysis. The median follow-up time for overall survival (OS) was 87.7 months. As per inclusion criteria, DTCs were detected in all patients. Patient characteristics are presented in detail in Table 1. The median age at diagnosis was 61.0 years (Q1–Q3: 50.0–68.0 years). The majority of patients were postmenopausal (n = 137, 68.2%). Most tumors were categorized as no special type (NST) (144, 71.6%), were grade 2 or grade 3 (n = 175, 87.9%)), and were sized <10 mm (n = 121, 60.2%). The majority was nodal negative (n = 128, 63.7%) and estrogen-receptor-positive (n = 166, 83.0%). There was no significant association between the HER2 status of DTCs and any of these clinicopathological factors.

### 2.2. HER2 Status of PT and DTCs

**Figure 1:**
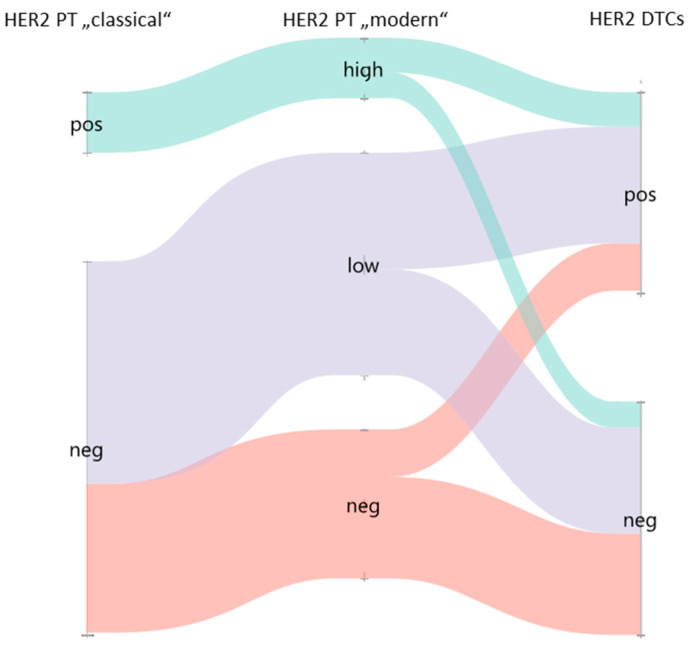
Sankey diagram of HER2 classification of the primary tumor (“classical” and “modern *”) and of HER2 status of DTCs. * modern: HER2-low was defined as HER2 IHC 1+ or IHC 2+ without FISH or CISH amplified, HER2-high as HER2 IHC 3+ or IHC 2+ with FISH or CISH amplified, and HER2-negative as HER2 IHC 0.

Of all patients, 93 (46%) had HER2-positive DTCs (Table 2). PTs classified as HER2-low or HER2-high were significantly more often associated with HER2-positive DTCs than HER2-zero PT (52.4% and 57.1% vs. 31.9%, *p* = 0.012, see Table 2). Concordance between the “modern” HER2 classification of PTs and HER2 status of DTCs is described in Table 2 and Figure 1.

A total of 28 PTs (13.9%) were classified as HER2-positive in the “classical” classification, meaning HER2 IHC 3+ or IHC 2+ with FISH or CISH amplified. No significant association was found for the HER2 status of PTs with the HER2 status of DTCs following the “classical” classification. Furthermore, a discordance of 44.3% was found between those two parameters (Table 2). When considering the “classical” HER2 classification, 59.9% of HER2-negative PTs were redefined as HER2-low (see Figure 1).

### 2.3. Systemic Therapy and HER2 Status of DTCs

Information on systemic treatment was available for 17 of the 28 patients with HER2-positive PTs. Of those, 15 received trastuzumab-based treatment, while 2 patients did not receive trastuzumab-based treatment.

Of the 15 patients with HER2-positive PTs who received trastuzumab-based treatment, no significant difference was found in regard to the HER2 status of DTCs (n = 5 with HER2-negative vs. n = 10 with HER2-positive DTCs, *p* = 0.768). Additionally, six of those patients were treated in a neoadjuvant setting; among the patients treated in a neoadjuvant setting, BM aspiration was performed in n = 2 after neoadjuvant treatment. No significant difference in HER2 status between the PT and DTCs was found when considering the timing of the treatment (neoadjuvant vs. adjuvant) or timing of BM aspiration (before or after neoadjuvant treatment).

### 2.4. Survival Analysis

Follow-up data were available in 201 cases for OS and CSS as well as DFS and for 200 cases for distant disease-free survival (dDFS). At a median follow-up of 87.7 months for OS, 19 patients had died of any cause, while 17 deaths were attributed to breast cancer. At a median follow-up of 77.0 months for DFS, 29 patients were suffering from recurrent disease and 24 patients had metastatic disease.

**Figure 2:**
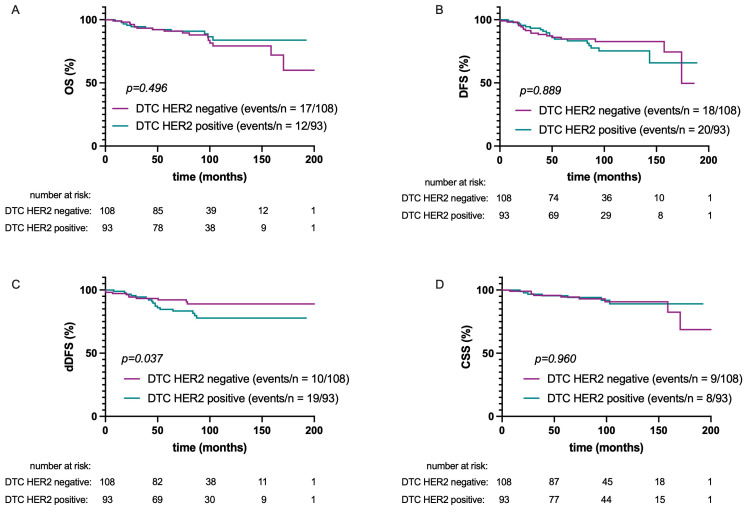
Kaplan–Meier plots of overall survival (OS) (**A**), disease-free survival (DFS) (**B**), distant disease-free survival (dDFS) (**C**), and cancer-specific survival (CSS) (**D**) according to HER2 status of DTCs.

The HER2 status of DTCs did not influence DFS, CSS, or OS (*p* = 0.889, *p* = 0.960, and *p* = 0.496, respectively; see Figure 2). A significant difference in dDFS was found according to DTC-HER2 status: dDFS was lower in patients whose DTCs were HER2-positive (HR: 2.3; 95% CI: 1.1–5.2; *p* = 0.037).

**Figure 3:**
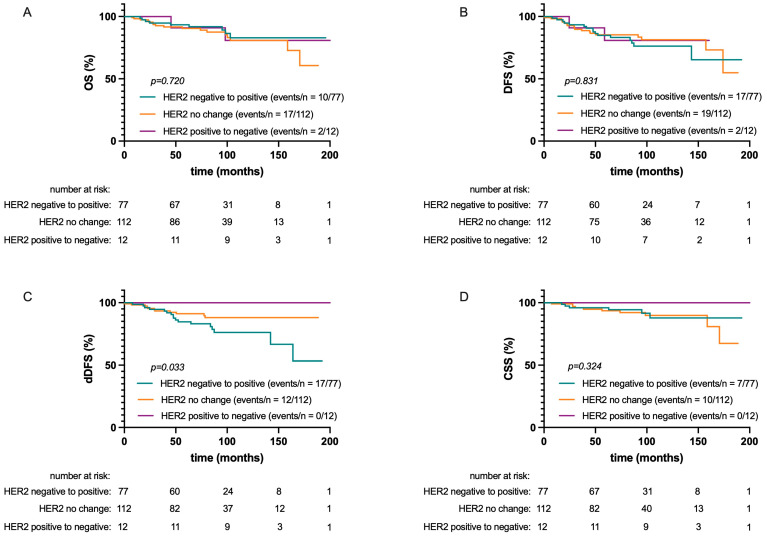
Kaplan–Meier plots of overall survival (OS) (**A**), disease-free survival (DFS) (**B**), distant disease-free survival (doffs) (**C**), and cancer-specific survival (CSS) (**D**) according to consistency of HER2 status between PT and DTCs.

As decisions on systemic therapy were based on the “classical” HER2 classification, homogeneity or discrepancies between the HER2 status of the PT and DTCs and their possible association with survival did not consider HER2-low as a distinct entity. No significant association was found between those parameters and OS (*p* = 0.720), CSS (*p* = 0.324), or DFS (*p* = 0.831; see Figure 3).

However, dDFS was significantly associated with consistency or inconsistency between the HER2 status of the PT and DTCs (*p* = 0.033). The worst dDFS was seen in patients with HER2-negative PT/HER2-positive DTCs compared to patients with HER2-positive PT/HER2-negative DTCs or concordant HER2 status between PT and DTCs (HR: 2.3; 95% CI: 1.1–4.9).

In the multivariate analysis (Table 3), independent factors of dDFS were estrogen receptor status, nodal status, HER2 status of PT, and HER2 status of DTCs. Independent factors of DFS were estrogen receptor status, nodal status, and tumor size. Finally, independent factors of OS were nodal status and estrogen receptor status.

## 3. Discussion

To the best of our knowledge, this is the first study to correlate the HER2 status of the PT following the modern interpretation of HER2 classification with the HER2 status of DTCs.

Previous studies found a discordance between the HER2 status of the PT and DTCs in 29–49% of cases [20,25,26,27]. These results are in line with the 44% discordance found in the present study when solely differentiating between “HER2-positive” and “HER2-negative”. However, when comparing the HER2 status of DTCs with the “modern” HER2 classification for the PT, many discordant cases are attributed to HER2-low PTs, with 52.4% of patients with HER2-low PTs showing HER2-positive DTCs. Therefore, when compared to the “modern” classification, a significant association between the HER2 status of the PT and of DTCs could be detected (*p* = 0.012).

A plausible explanation for the newly detected correlation between an HER2-low PT and HER2-positive DTCs may be the lack of a threshold for differentiating between HER2-low or HER2-high expression in single-cell analysis of DTCs in comparison to the HER2 scoring system in PT tissue [28].

The presence of DTCs in the BM of breast cancer patients was previously shown to be associated with a poorer prognosis [8,9,10,29,30]. When further analyzing DTCs, it could also be demonstrated that molecular features such as the HER2 status of DTCs can further stratify the risk of disease recurrence in EBC [20,30]. In the present study, similar findings could be reported, where adverse dDFS could be seen in patients whose DTCs were HER2-positive (HR: 2.3; 95% CI: 1.1–5.2; *p* = 0.037). The influence of the HER2 status of DTCs on dDFS was further confirmed in the multivariate analysis (HR 2.6, 95% CI 1.2–5.9, *p* = 0.021).

More interestingly, dDFS was also associated with a discrepancy between the HER2 status of the PT and DTCs (*p* = 0.033): patients with HER2-negative PT/HER2-positive DTCs had worse outcomes than those with HER2-positive PT/HER2-negative DTCs or concordant HER2 status (HR: 2.3; 95% CI: 1.1–4.9). Similar results could be seen in patients with advanced breast cancer and discordant HER2 status between the PT and circulating tumor cells (CTCs) isolated from peripheral blood [31], where disease progressed more quickly in patients with an HER2-negative PT and HER2-positive CTCs [32,33].

It was previously shown that HER2-positive single cells or cell subpopulations found in breast PTs have a higher tendency to disseminate [34]. These results correlate with the results of the present study, where HER2 expression in DTCs was associated with a higher tendency for distant metastases. Breast carcinomas in which HER2 was overexpressed were found to have a different metastatic pattern associated with higher aggressivity [35]. In addition, HER2 overexpression was found to activate cellular signaling pathways, which seem to activate a phenotype more prone to dissemination [36].

The study results indicate a poorer outcome for patients in whom HER2-negative is converted to positive between the PT and DTCs. This highlights the importance of distinguishing between the characteristics of PTs and micrometastases when making therapeutic decisions. Administration of trastuzumab for HER2-negative PTs but HER2-positive CTCs was associated with an improved prognosis in gastric cancer [37]. However, trials in metastatic breast cancer, in which patients with HER2-positive CTCs received trastuzumab or trastuzumab emtansine, did not show benefits for those patients. It is important to note, however, that these trials had limited patient numbers and further research is needed to confirm these findings [38,39].

One major limitation of this study is its retrospective character and the lengthy period of data collection given the evolution of therapeutic approaches over time, particularly in recent years. However, over 200 patients were included in this study and it covered a median follow-up time of over 7 years for OS, which represent strengths of the work. The survival models in the present study convey an impact of the HER2 status of the PT and DTCs on metastatic-free survival; however, no effect was found in OS and CSS. This could be attributed to the increasing emergence of HER2-targeting therapies in the last decade.

Further trials should validate the impact of the HER2 status of DTCs in patients receiving modern systemic therapy for EBC. Prospective trials could then focus on adapting therapy to the HER2 status of micrometastases. With the implementation of new HER2-targeting therapies for breast cancer [40] and the emergence of trials investigating the antibody drug conjugate trastuzumab deruxtecan targeting HER2-low EBC [41], similar analyses to those in the present study should be conducted to evaluate potential effects of new HER2 therapies on micrometastases and their impact on survival.

In conclusion, this trial showed a significant association of HER2-low PTs with HER2-positive DTCs. Discrepancies between PTs and DTCs may indicate a conversion of HER2 status during micrometastatic processes in EBC. Indeed, these discrepancies were associated with the occurrence of distant metastases, which raises questions about the necessity of specific systemic treatments adapted to micrometastasis.

## 4. Materials and Methods

### 4.1. Study Population

Women with EBC undergoing surgery at the Department of Obstetrics and Gynecology, University of Tuebingen, Germany, between January 2001 and December 2017 were eligible for this study. Only patients in whom DTCs were detected in the BM were included (see method below). Exclusion criteria were bilateral breast cancer, metastatic or recurrent disease, R1 resection, and previous cancer of other origin. All patients provided written informed consent, and the analysis was approved by the local ethics committee (reference number: 528/2019BO2).

Systemic therapy was based on current St. Gallen recommendations and national treatment guidelines [42,43,44].

### 4.2. Detection and Characterization of DTC

BM was sampled during surgery. Written consent for BM sampling and for BM and data processing was given prior to the operation. All BM samples were processed within 24 h. Mononuclear cells from the BM were isolated by density centrifugation (Ficoll, 1.077 g/mL, Biochrom, Berlin, Germany). These cells were then spun down onto a glass slide (cytocentrifuge, Hettich, Tuttlingen, Germany) and fixed in 4% formalin. The obtained cytospins were stained using the DAKO Autostainer (DAKO, Glostrup, Denmark). Mouse monoclonal antibodies A45-B/B3 directed against pancytokeratin (Micromet, Munich, Germany) and keratin 8/18 Ab-1 (Thermo Fisher Scientific, Fremont, CA, USA) and mouse monoclonal antibody directed against c-erbB-2 (Her-2/neu) (BioGenex, Fremont, CA, USA) were used. For cytokeratin staining, two slides with 1.5 × 10^6^ cells per patient were evaluated, according to the consensus recommendations for standardized tumor cell detection [13]. DTC positivity was defined as at least one pancytokeratin-positive cell with typical cell morphology [45] per 1.5 × 10^6^ cells. For HER2 staining, one slide with 1.5 × 10^6^ cells per patient was evaluated and HER2 positivity was defined as one HER2-positive cell with typical cell morphology. Each batch of samples was analyzed together with leukocytes from healthy volunteers as negative controls and the human breast cancer cell lines MCF 7 and SKBR 3 as positive controls for pancytokeratin and HER2 staining.

### 4.3. Evaluation of the HER2 Status of the PT

HER2 status of the PT was determined using the HERCEP™ immunochemistry test (DAKO, Glostrup, Denmark). Expression of HER2 was scored on a scale from 0 to +3. Two classifications for HER2 status of the PT were applied, and these were further defined as the “classical” and the “modern” classification. For the “modern” HER2 classification, tumors with a score of 0 were considered HER2-zero, while those with a score of +3 were considered HER2-high. For scores of +2, HER2 amplification was determined by fluorescence in situ hybridization (FISH) using the Pathvysion^®^ Kit (Vysis, Downers Grove, IL, USA). Tumors with a score of +2 and FISH amplification were also considered HER2-high. Finally, those with +2 and negative FISH amplification or a score of +1 were defined as HER2-low. For the “classical” HER2 classification, tumors with a score of +3 were considered HER2-positive. For scores of +2, HER2 amplification was determined by fluorescence in situ hybridization (FISH) using the Pathvysion^®^ Kit (Vysis, Downers Grove, IL, USA). Tumors with a score of +2 and FISH amplification were also considered HER2-positive, while those with +2 and negative FISH amplification and those with a score of 0 or +1 were defined as HER2-negative.

### 4.4. Statistical Analysis

Associations between categorical variables were analyzed using the chi-squared test and Fisher’s exact test. Associations between continuous variables were analyzed using Student’s *t*-test. For survival analysis, time from BM aspiration to any recurrence of disease (DFS) and to death of any cause (overall survival, OS) was investigated separately. For disease recurrence, we further distinguished between locoregional recurrence and distant metastasis. For the latter, time from BM aspiration to metastasis (distant DFS, dDFS) was calculated. Further, time from BM aspiration to death from breast cancer (cancer-specific survival, CSS) was investigated. If no event had occurred, data were censored at last follow-up. For univariate analysis of the influence of DTC status and DTC-HER2 status on survival, Kaplan–Meier curves were plotted and compared using the log-rank test. For multivariate analysis, a Cox proportional hazard regression model was used to assess the simultaneous effect of different factors on survival. A significance level of 0.1 in the univariate analysis was used to exclude a variable from the model. The initial model included menopausal status, histology, grading, tumor size, nodal status, estrogen receptor status, HER2 status, and DTC-HER2 status. The effect of each variable was evaluated using likelihood ratio testing and described by hazard ratio (HR) and the corresponding 95% confidence interval (CI). All statistical analyses were performed using JMP16 (SAS^®^, Heidelberg, Germany). Significance level was set at *p* < 0.05.

## Figures and Tables

**Table 1:** Patient characteristics in regard to DTC-HER2 status.

	All Patients n	DTC HER2-Positive n (%)	*p*-Value *
	201	93 (46.2)	
Mean age at diagnosis (years)	59.9	60.0	0.891
Menopausal status			0.544
premenopausal	64	32 (50.0)	
postmenopausal	137	61 (44.5)	
Histological type			
NST	144	70 (49.6)	0.058
ILC	39	12 (30.8)	
other	18	11 (61.1)	
pT			0.948
T1	121	56 (46.3)	
T2–4	80	37 (46.3)	
pN			
N0	128	59 (46.1)	1.000
N+	73	34 (46.6)	
ER status			
positive	166	75 (45.2)	0.706
negative	34	17 (50.0)	
Grading			
G1	24	11 (45.8)	0.968
G2–3	175	81 (46.3)	

* Fisher’s test was used for categorical variables; *t*-test was used for continuous variables (age).

**Table 2:** Association between HER2 classification of PT and HER2 status of DTCs.

Patients HER2 Classification	Total n	DTC HER2-Positive n (%)	DTC HER2-Negative n (%)	*p*-Value *
HER2 “classical”				0.214
Negative	173	77 (44.5)	96 (55.5)	
Positive	28	16 (57.1)	12 (42.9)	
HER2 “modern”				
Negative	69	22 (31.9)	47 (68.1)	0.012
Low	103	54 (52.4)	49 (47.6)	
High	28	16 (57.1)	12 (42.9)	

* chi-squared test.

**Table 3:** Multivariate regression analysis of dDFS, DFS, OS, and CSS.

Parameter	dDFS				DFS				OS				CSS			
	Univariate		Multivariate		Univariate		Multivariate		Univariate		Multivariate		Univariate		Multivariate	
	HR (95% CI)	*p*-Value	HR (95% CI)	*p*-Value	HR (95% CI)	*p*-Value	HR (95% CI)	*p*-Value	HR (95% CI)	*p*-Value	HR (95% CI)	*p*-Value	HR (95% CI)	*p*-Value	HR (95% CI)	*p*-Value
Estrogen receptor status																
neg. vs. pos	3.8 (1.8–8.2)	0.001	15.7 (5.6–44.1)	<0.001	3.4 (1.7–6.7)	<0.001	3.8 (1.9–7.5)	<0.001	1.8 (0.8–4.1)	0.034	2.6 (1.1–6.3)	0.036	3.5 (1.3–9.3)	0.015	19.4 (4.9–77.2)	<0.001
Nodal status																
pos. vs. neg.	4.5 (2.0–10.2)	<0.001	9.8 (3.3–28.9)	<0.001	3.3 (1.7–6.4)	<0.001	3.0 (1.4–6.1)	0.003	3.0 (1.4–6.4)	0.004	4.5 (1.9–10.5)	<0.001	4.9 (1.7–14.3)	0.003	17.2 (3.7–79.4)	<0.001
HER2 status PT																
pos. vs. neg.	2.7 (0.6–11.4)	0.032	12.9 (2.4–48.7)	0.003	1.1 (0.4–2.8)	0.087		0.191	1.3 (0.4–3.8)	0.061		0.111	3.6 (0.5–28.4)	0.097	34.9 (3.1–397.6)	0.004
HER2 status DTCs																
pos. vs. neg.	2.3 (1.1–5.2)	0.037	2.6 (1.2–5.9)	0.021	1.2 (0.6–2.2)	0.064		0.837	1.3 (0.6–2.7)	0.094		0.346	1.0 (0.4–2.7)	0.096		0.934
Tumor size																
T1 vs. T2–4	4.0 (1.8–8.8)	<0.001		0.062	3.3 (1.7–6.5)	<0.001	0.5 (0.22–0.98)	0.04	2.3 (1.1–4.9)	0.022		0.114	2.7 (1.0–7.1)	0.047		0.613

## Data Availability

The data presented in this study are available on request from the corresponding author.
